# Population genetic analysis and sub-structuring of *Theileria parva* in the northern and eastern parts of Zambia

**DOI:** 10.1186/1756-3305-5-255

**Published:** 2012-11-12

**Authors:** Walter Muleya, Boniface Namangala, Martin Simuunza, Ryo Nakao, Noboru Inoue, Takashi Kimura, Kimihito Ito, Chihiro Sugimoto, Hirofumi Sawa

**Affiliations:** 1 Division of Molecular Pathobiology, Research Center for Zoonosis Control, Hokkaido University, N20, W10, Kita-ku, Sapporo, 001-0020, Japan; 2 Department of Biomedical Sciences, School of Veterinary Medicine, University of Zambia, P.O Box 32379, Lusaka, 10101, Zambia; 3 Department of Para-clinical Studies, School of Veterinary Medicine, University of Zambia, P.O Box 32379, Lusaka, 10101, Zambia; 4 Department of Disease Control, School of Veterinary Medicine, University of Zambia, P.O Box 32379, Lusaka, 10101, Zambia; 5 Division of Collaboration and Education, Research Center for Zoonosis Control, Hokkaido University, N20, W10, Kita-ku, Sapporo, 001-0020, Japan; 6 National Research Center for Protozoan Diseases, Obihiro University of Agriculture and Veterinary Medicine, Inada-cho, Hokkaido, 080-8555, Japan; 7 Division of Bioinformatics, Research Center for Zoonosis Control, Hokkaido University, N20, W10, Kita-ku, Sapporo, 001-0020, Japan; 8 Global COE program, Research Center for Zoonosis Control, Hokkaido University, N20, W10, Kita-ku, Sapporo, 001-0020, Japan

**Keywords:** *Theileria parva*, Genetic diversity, Sub-structuring, Zambia

## Abstract

**Background:**

Theileriosis, caused by *Theileria parva*, is an economically important disease in Africa. It is a major constraint to the development of the livestock industry in some parts of eastern, central and southern Africa. In Zambia, theileriosis causes losses of up to 10,000 cattle annually.

**Methods:**

Cattle blood samples were collected for genetic analysis of *Theileria parva* from Isoka and Petauke districts in Zambia. Microsatellite analysis was then performed on all *Theileria parva* positive samples for PCR using a panel of 9 microsatellite markers. Microsatellite data was analyzed using microsatellite toolkit, GenAlEx ver. 6, Fstat ver. 2.9.3.2, and LIAN computer softwares.

**Results:**

The combined percentage of positive samples in both districts determined by PCR using the p104 gene primers was 54.9% (95% CI: 46.7 – 63.1%, 78/142), while in each district, it was 44.8% (95% CI: 34.8 – 54.8%) and 76.1% (95% CI = 63.9 – 88.4%) for Isoka and Petauke districts, respectively. We analyzed the population genetic structure of *Theileria parva* from a total of 61 samples (33 from Isoka and 28 from Petauke) using a panel of 9 microsatellite markers encompassing the 4 chromosomes of *Theileria parva*. Wright’s F index (F_ST_ = 0.178) showed significant differentiation between the Isoka and Petauke populations. Linkage disequilibrium was observed when populations from both districts were treated as a single population. When analyzed separately, linkage disequilibrium was observed in Kanyelele and Kalembe areas in Isoka district, Isoka district overall and in Petauke district. Petauke district had a higher multiplicity of infection than Isoka district.

**Conclusion:**

Population genetic analyses of *Theileria parva* from Isoka and Petauke districts showed a low level of genotype exchange between the districts, but a high level of genetic diversity within each district population, implying genetic and geographic sub-structuring between the districts. The sub-structuring observed, along with the lack of panmixia in the populations, could have been due to low transmission levels at the time of sampling. However, the Isoka population was less diverse than the Petauke population.

## Background

Theileriosis is an economically important disease of cattle in eastern, central and southern Africa. The disease, caused by the protozoan haemoparasite *Theileria parva* (*T. parva*), is transmitted by the 3-host tick *Rhipicephalus appendiculatus*[1]. The severity of the disease manifests differently in various breeds of cattle, with the zebu cattle (*Bos indicus*) being more resistant than the exotic breeds (*Bos taurus*) against both the parasite [2] and the vector [3,4]. Theileriosis causes high levels of mortality in *taurine* breeds [5] and both high morbidity and mortality in indigenous 1–6 month old calves (*Bos indicus*). Full-scale epidemics affecting all age groups of indigenous breeds may occur [6], resulting in reduction in productivity. The high level of mortality in *taurine* breeds impedes the introduction of highly productive exotic breeds in endemic areas, further preventing the improvement of cattle production in the affected areas. Theileriosis is thus a major constraint to the development of the livestock industry in the eastern, central and southern parts of Africa.

**Figure 1:**
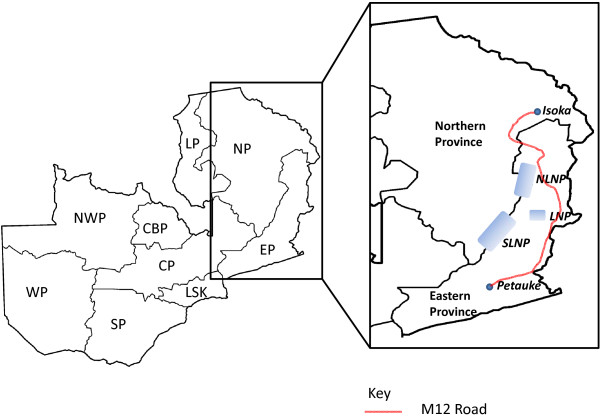
**Map of the Republic of Zambia showing its nine [**[9]**] provinces namely; the Northern (NP), Eastern (EP), Luapula (LP), Central (CP), Copperbelt (CBP), Lusaka (LSK) Southern (SP), Western (WP) and North-western (NWP) provinces.** The *Theileria parva* samples used in this study were obtained from Isoka (NP) and Petauke (EP) districts. Isoka district is separated from Petauke district by the North Luangwa National Park (NLNP), Lukusuzi National Park (LNP), and the South Luangwa National Park (SLNP) and connected by the M12 commercial road.

The republic of Zambia, divided into 9 provinces for administrative purposes, is located in south-central Africa. In Zambia, theileriosis is referred to as East Coast fever (ECF) in the northern and eastern provinces (NP and EP) and Corridor Disease (CD) in the southern, central, lusaka and copperbelt provinces (SP, CP, LSK and CBP) (Figure 1) [7]. Theileriosis causes losses of up to 10,000 cattle annually and approximately 1.4 million of the 3 million cattle population is at risk of theileriosis [8]. Theileriosis control in Zambia is based on a combined system of (i) vector control (using acaricides), (ii) cattle movement control, (iii) chemotherapy, and (iv) immunization (infection and treatment) using the Katete (EP) and Chitongo (SP) stocks [9].

*T. parva* is mainly haploid during its life cycle, except for the polyploidy schizont stage and the diploid zygote and kinete stages [10]. Briefly, *T. parva* male and female gametes in the tick gut fuse to form short-lived diploid zygotes which then develop into motile kinetes [11] and migrate to the tick salivary glands where they divide meiotically into sporozoites [10,12]. The sporozoites, once introduced into cattle by the feeding tick, rapidly invade leucocytes, differentiating into intralymphocytic schizonts with a certain proportion undergoing merogony giving rise to uni-nucleate merozoites [10,13]. The rupture of lymphocytes liberates the merozoites into the blood stream where they develop into intraerythrocytic piroplasms, the stage that is ingested by the tick vector during a blood meal [10,13]. Genetic exchange has been shown to occur when 2 or more stocks of *T. parva* are used to infect the tick vector [14].

Studies on the population structures of various protozoan parasite species [15-21] have shown clonal (*Trypanosoma cruzi*, *Toxoplasma gondii* and *Cryptosporidium hominis*) [22-24], panmixic (*Cryptosporidium parvum*) [24] and epidemic (*Crytosporiduim parvum*) [25] population structures. On the other hand, *Trypanosoma brucei* and *Plasmodium falciparum* population structures have shown dependence on host specificity and transmission intensity [26,27]. In general, the conclusion from these studies is that despite the presence of an obligatory sexual phase in their life cycle, a number of factors determine the population structures of different protozoan parasitic species.

Micro- and mini-satellite markers have been used to genotype several species of apicomplexan parasites and their vectors, revealing different population structures [18,21,26-30]. For example, a study on the population structure of *T. parva* in Uganda reported a mixture of genotypes in many isolates and linkage disequilibrium (LD) in 3 populations isolated from different areas [18]. When multiple isolates with identical genotypes were treated as a single isolate, the presence of an epidemic structure was seen in 2 of the populations, suggesting an intermediate between extreme clonality and panmixia [18].

A crucial and important aspect in population genetic studies is to determine the effect of random and non-random mating on the population structures of disease-causing agents and consequently on the epidemiology of the diseases [31]. Information on the genetic exchange in *T. parva* populations has practical implications in disease control and prevention. For instance, populations with a high degree of genetic diversity arise when high levels of recombination occur. This information is very important in vaccine development as it is easier to develop a vaccine against a clonal pathogen than a highly diverse pathogen. A panel of 9 polymorphic micro-satellite markers was used to genotype *T. parva* positive cattle blood DNA in order to answer the following questions: (i) what is the population structure of the *T. parva* in eastern and northern Zambia? (ii) Does gene flow occur between *T. parva* populations sampled from each area? (iii) Do the *T. parva* populations from the sampling areas consist of a single or multiple distinct populations? To our knowledge, this is the first study on the population genetics of *T. parva* in Zambia.

## Methods

### Sample collection and DNA preparation

This study received ethical clearance for collection of animal blood from the Biomedical Research Ethics Committee, University of Zambia and from the Department of Veterinary and Livestock Development, Eastern Province, Zambia. The recombinant DNA experiments were approved by Hokkaido University.

About 10 mL of whole blood samples (n=142) were collected in heparinized tubes from indigenous and mixed breeds of cattle from Kanyelele (n = 62) and Kalembe (n=34) areas in Isoka district (n=96) of the northern province (NP) and from Saukani area in Petauke district (n=46) of the eastern province (EP) (Figure 1) of Zambia in May 2008, after the wet season. Kanyelele is located approximately 20 km from Kalembe. Isoka and Petauke districts are approximately 600 km apart (straight line distance) and are separated by the North Luangwa, South Luangwa and Lukusuzi National Parks (NLNP, SLNP and LNP, respectively, in Figure 1). Petauke and Isoka districts are connected by a commercial road (Figure 1) covering a distance of about 795 km. Whole genome DNA was extracted using DNAzol (Molecular Research Center, Cincinnati, OH) following the manufacturer’s instructions and stored at −20°C. A panel of 16 polymorphic microsatellite markers [32-34], representing the 4 chromosomes, was initially selected (Table 1) for genotyping of the samples. The following *T. parva* parasite laboratory stocks were used for the initial marker screening to determine which markers amplified more isolates and were sufficiently polymorphic for use in the genotyping of field samples from Isoka and Petauke districts: Onderstepoort (South Africa, year of isolation unknown), Serengeti (Tanzania, 1978), Muguga (Kenya, year of isolation unknown), Nyakizu (Rwanda, 1979), Entebbe (Uganda, 1980), Katumba (Burundi, 1981), Kiambu Z464/C12 and B8 (Kenya, 1972), Katete B2 (Zambia, 1989) and Buffalo Z5E5 (both origin and year of isolation unknown). These stocks were also used as Genescan control samples during the analysis of field samples.

**Table 1:** **Panel of microsatellite markers used to genotype *Theileria parva *samples from Isoka and Petauke districts **

**Marker**	**Chromosome**	**Amplicon size (bp)**	**Used in final analysis**	**Reference**
MS1	1	235-368	No	Oura *et al*., 2003 [32]
MS62	1	271	No	Katzer et al., 2010 [34]
**MS48**	**1**	223	**Yes**	Katzer et al., 2006 [33]
MS5	1	206-444	No	Oura et al., 2003 [32]
MS66	1	266	No	Katzer et al., 2010 [34]
MS67	1	245	No	Katzer et al., 2010 [34]
MS77	2	270	No	Katzer et al., 2010 [34]
**MS71**	**2**	252	**Yes**	Katzer et al., 2010 [34]
**MS74**	**2**	246	**Yes**	Katzer et al., 2010 [34]
**MS75**	**2**	244	**Yes**	Katzer et al., 2010 [34]
**MS72**	**2**	230	**Yes**	Katzer et al., 2010 [34]
**MS51**	**3**	161	**Yes**	Katzer et al., 2006 [33]
**MS53**	**3**	208	**Yes**	Katzer et al., 2006 [33]
**MS57**	**4**	118	**Yes**	Katzer et al., 2006 [33]
MS58	4	300	No	Katzer et al., 2006 [33]
**MS59**	**4**	111	**Yes**	Katzer et al., 2006[33]

### *T. parva* screening

Whole blood DNA field samples were screened for *T. parva* DNA by using *T. parva*-specific p104 gene primers [35]. Template DNA (1μL) was amplified in a 20 μL reaction mixture as prescribed by the manufacturer using ExTaq polymerase (Takara, Tokyo, Japan). The PCR conditions were as follows: denaturation at 95°C for 5 minutes; 35 cycles at 95°C for 1 minute, 63°C for 30 seconds and 72°C for 1 minute, followed by a final extension step of 5 minutes at 72°C. The amplified products were analyzed on 2% ethidium bromide pre-stained agarose gel.

### PCR amplification and analysis of microsatellite loci

PCR was performed using primers (Table 1) designed to amplify each of the 16 repeat regions on each of the *T. parva* stocks isolated from different geographical areas. The forward primer in each primer marker set was fluorescently labeled. The 20 μL PCR mixture used comprised of 10 ng of template DNA, 10 μL of 2x Amplitaq Gold master mix (Invitrogen, CA), 0.25 μM of each primer and distilled water. The PCR conditions were as follows: denaturation at 95°C for 5 minutes; 35 cycles at 95°C for 15 seconds, 50 - 58°C for 60 seconds and 72°C for 1 minute, followed by a final extension step of 5 minutes at 72°C. The amplified products were observed on 1.5% ethidium bromide pre-stained agarose gels to determine the success of PCR amplification. To achieve high genotyping resolution of field samples, microsatellite PCR products were denatured and then capillary electrophoresed in an ABI 3130 genetic analyzer (Applied Biosystems, CA). DNA fragment sizes were analyzed relative to the ROX-labeled GS600 LIZ size standard (Applied Biosystems) using Gene Mapper software (Applied Biosystems). This facilitated the resolution of multiple products with 1 base pair (bp) difference in a single reaction. Multiple products from a single PCR reaction indicated the presence of mixed genotypes. The output data from the genetic analyzer were provided as the area under the peak of each allele (quantitative measurement), with the predominant allele possessing the greatest peak area. In this way, the predominant allele at each locus was identified for each sample, and this data was combined to generate a multi-locus genotype (MLG) representing the most abundant genotype in each sample. Only the alleles with the prescribed base pair range were used to generate the MLG and samples from the same area were electrophoresed and genescaned on the same plate. Separate PCRs were also carried out for different regions.

### Data analysis

An allele sharing co-efficient [36] in Excel microsatellite toolkit (http://animalgenomics.ucd.i.e./sdepark/ms-toolkit/) was used for the similarity comparison of the MLGs [37]. Principal component analysis (PCA) was used for similarity analysis. A similarity matrix was constructed and used to construct PCA using the Excel plug-in software GenAIEx6 (http://www.anu.edu.au/BoZo/GenAIEx/) [38]. F_STAT_ computer package version 2.9.3.2 was used to calculate estimates of F statistics for population genetic analysis (http://www2.unil.ch/popgen/softwares/fstat.htm). LIAN (http://adenine.biz.fh-weihenstephan.de/lian/) was used to test the null hypothesis of linkage equilibrium by calculating a quantification of linkage equilibrium/linkage disequilibrium called the standardized index of association (*I*_*A*_^*S*^) [39]. The statistical independence of alleles at all pairwise combinations of loci under study characterizes linkage equilibrium (LE) and this independent assortment was initially tested by LIAN by determining the number of loci at which each pair of MLGs differs. The mis-match values from this distribution were then used to calculate the variance (V_D_) which was then compared to the variance expected (V_E_) for LE. Monte Carlo (MC) computer simulation was used to test the null hypothesis that V_D_ = V_E_. The computer software calculates a 95% confidence limit L (L_MC_). When V_D_ is greater than L, the null hypothesis of LE is discarded.

### Multiplicity of infection

The majority of the samples in this study possessed several alleles at one or more loci, representing a mixed infection. The mean number of alleles for the nine loci in each sample was calculated and this index value represented the multiplicity of infection within each sample. The overall mean for the index values for each sample was then calculated to provide the multiplicity of infection for each region.

## Results

### PCR screening

The screening of cattle blood samples (n=142) using the p104 gene primers showed a combined *T. parva* positive percentage of 54.9% (95% CI: 46.7–63.1%, 78/142) from both districts. The percentage of positive samples within each district was 44.8% (95% CI: 34.8–54.8%) (23 from Kanyelele and 20 from Kalembe, 43/96) and 76.1% (95% CI: 63.9–88.4%, 35/46) for Isoka and Petauke (Saukani area), respectively.

### Marker diversity and allelic variation

A panel of 9 polymorphic microsatellite markers out of the initial 16 markers (Table 1), representing the 4 chromosomes of *T. parva*, was used to genotype 61 *T. parva* positive samples, that is 33 (14 from Kalembe and 19 from Kanyelele areas) from Isoka district and 28 from Petauke district (Saukani area). Seven markers that either failed to produce signals on gel electrophoresis or exhibited reduced polymorphism were excluded from the final analysis (Table 1). Seventeen samples were excluded from the final analysis because most of the markers failed to produce signals in them on gel electrophoresis. The reason for the failure of amplification of the target regions in these samples (n=17) was probably due to the poor quality of the DNA.

The maximum number of alleles identified by each marker in each population ranged from 3 (MS72) to 12 (MS71) for Isoka and 4 (MS57 and MS75) to 12 (MS53) for Petauke (Table 2). Marker MS53 was the most polymorphic, identifying 19 alleles in both populations whereas MS57 was the least polymorphic, identifying only 6 alleles. Similar gene diversities across all loci were observed between Isoka and Petauke populations except for markers MS48, MS59 and MS72 that exhibited slightly larger differences between the 2 populations (Table 2).

**Table 2:** **Allelic variation among *Theileria parva *from Petauke and Isoka districts of Zambia**

		**N**	**MS48**	**MS51**	**MS53**	**MS57**	**MS59**	**MS71**	**MS75**	**MS72**	**MS74**
Alleles within population	Petauke	28	7	6	12	4	6	6	4	6	9
	Isoka	33	5	8	7	5	5	12	5	3	11
	Overall	61	12	11	19	6	9	17	7	8	17
Gene diversity	Petauke	28	0.820	0.794	0.833	0.648	0.571	0.783	0.696	0.743	0.706
	Isoka	33	0.502	0.813	0.752	0.534	0.331	0.837	0.604	0.504	0.695

**Figure 2:**
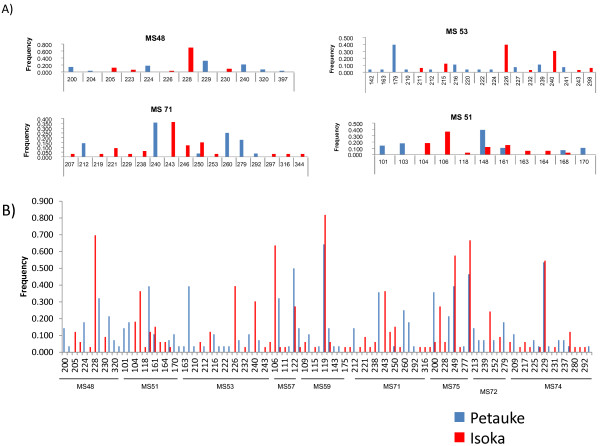
**The allele frequencies of alleles in field populations of *Theileria parva *from Petauke and Isoka districts of Zambia. ** The frequency of each predominant allele in the samples from the Petauke and Isoka populations was calculated and presented in the form of histograms. (**A**) The results for MS48 and MS53 show the presence of unique alleles and the absence of shared alleles. The remaining loci generally show a greater proportion of unique alleles as compared to shared alleles, as represented by MS 71 and MS 51. (**B**) The overall allele frequency in both populations shows a greater number of unique alleles and a reduced number of shared alleles. Multi-locus genotype (MLG) data was used to generate the histograms. The frequency of each predominant allele was calculated as a proportion of the total of each marker.

Allele frequencies at each locus for Isoka and Petauke populations were assessed with both populations showing high levels of diversity. Markers MS48 and MS53 showed only unique alleles in both populations (Figure 2A). Of the 12 alleles identified by MS48, 5 were from Isoka and 7 from Petauke, while out of the 19 alleles identified by MS53, 7 were from Isoka and 12 from Petauke (Figure 2A). The remaining markers showed both unique and shared alleles. For example, MS71 showed 11 and 5 alleles specific to Isoka and Petauke populations, respectively, with a single shared allele between Isoka and Petauke (Figure 2A). MS51 also showed 5 and 3 unique alleles specific to Isoka and Petauke, respectively, with 3 shared alleles (Figure 2A). Marker MS59 showed the lowest frequency of unique alleles (data not shown). A total of 91 (46 from Isoka and 45 from Petauke districts) unique alleles and 15 shared alleles were observed (Figure 2B).

### Similarity analysis between the Petauke and Isoka populations

**Figure 3:**
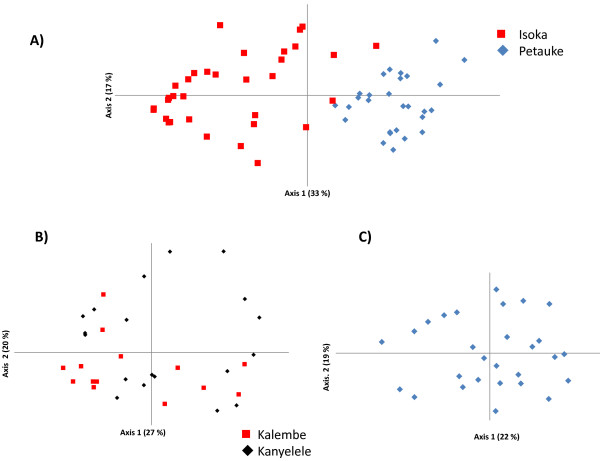
**Principal component analysis (PCA) of *T. parva *populations from Petauke and Isoka districts of Zambia. ** The proportion of variation in the population dataset explained by each axis is shown in parentheses. PCA was performed using multi-locus genotype data from Petauke and Isoka districts. (**A**) Geographical sub-structuring between populations from Petauke and Isoka districts. (**B**) Homogenous population from Isoka district with the Kanyelele population showing more diversity by occupying all 4 quadrants while the Kalembe population occupies only 3 quadrants. (**C**) Saukani population from Petauke district.

Evidence of sub-structuring was assessed by using PCA. Independent clustering of samples from Isoka and Petauke districts was observed, suggesting a state of geographical sub-structuring (Figure 3A). To determine the levels of sub-structuring on a finer scale, a separate PCA was constructed for samples from each district. Most of the samples from Isoka district, despite originating from 2 different areas (Kalembe and Kanyelele), occupied the same quadrants, except for four samples from Kanyelele which can be seen in the upper right quadrant of the PCA (Figure 3B). The samples from Petauke district (Saukani area) occupied all quadrants. Five samples in the upper left, 4 in the upper right and 2 in the lower left quadrants, appeared to exhibit some level of independence from the rest of the samples that had congregated mainly in the lower right quadrant, lower parts of the upper left and right quadrants and the left part of the lower left quadrant (Figure 3C) but due to their reduced number, further investigation into these possible differences was not carried out.

### Population diversity and differentiation

Estimated heterozygosities were calculated for samples from each district (Figure 3). The estimated heterozygosity (H_e_) and mean number of genotypes per locus for Kanyelele and Kalembe areas in Isoka were 0.675 and 5.67, and 0.503 and 3.67, respectively, with the population from Kanyelele being more diverse than that from Kalembe. The overall estimated heterozygosity (H_e_) and mean number of genotypes/locus for Isoka district was 0.619 and 6.78, respectively (Table 3). The H_e_ and mean number of genotypes/locus for Petauke district was 0.733 and 6.67, respectively (Table 3). The overall estimated heterozygosity for Petauke district showed that samples from this district were more diverse than those from Isoka (Table 3) despite both districts having a similar number of genotypes per locus. The overall heterozygosity for the two districts was 0.745 with a mean number of genotypes/locus of 11.78.

**Table 3:** **Population genetic analyses of *Theileria parva *from Petauke and Isoka districts of Zambia **

**Population**	**N**	**H**_**e**_	**Mean numbers genotypes/locus**	***I***_***A***_^***S***^	**V**_**D**_	**L**	**P-value**	**Linkage**	**F**_**ST**_
Between districts	61	0.745	11.78	0.078	2.457	1.677	< 0.001	LD	0.178
Isoka district									
Kanyelele area	19	0.675	5.67	0.075	2.637	2.066	< 0.001	LD	
Kalembe area	14	0.503	3.67	0.243	5.763	2.859	< 0.001	LD	
Isoka overall	33	0.619	6.78	0.104	3.418	2.254	< 0.001	LD	0.049
Petauke district									
Saukani area	28	0.733	6.67	0.027	1.979	1.910	0.015	LD	0.154

N: number of samples, H_e_: estimated heterozygosity, *I*_*A*_^*S*^: standard index of association, V_D_: mismatch variance (linkage analysis), L: upper 95% confidence limit of Monte Carlo simulation (linkage analysis) and LD: linkage disequilibrium and LE: linkage equilibrium.

To measure the levels of genetic differentiation, Wright’s F index was calculated between Isoka district and Petauke district populations, for the Isoka sub-populations and also within Petauke district (Table 3). An F_ST_ value of 0.178 was observed between Isoka and Petauke district populations (Table 3), showing significant genetic differentiation. The F_ST_ value for the Isoka populations (Kanyelele and Kalembe) was 0.049, implying moderate differentiation between the 2 populations (Table 3). The F_ST_ value for Petauke (Figure 3C) was 0.154, showing significant differentiation.

### Genetic analysis

In order to determine whether the parasite populations within the two districts comprised a single panmictic population with a high degree of genetic exchange, the level of LE of the alleles at pairs of loci was measured using the standard index of association (*I*_*A*_^*S*^). The *I*_*A*_^*S*^ measures the association between alleles at pairs of loci, with *I*_*A*_^*S*^ values close to 0 or negative indicating panmixia and those significantly greater than 0 indicating non-panmixia [39]. The variance of pairwise differences (V_D_) between the data and that predicted for panmixia (V_E_) and L, the 95% confidence interval for V_D_ relative to the null hypothesis, were calculated in order to test the hypothesis of panmixia. Using this analytical method, when the V_D_ value exceeds the L value, LD is indicated and the null hypothesis of panmixia is discarded. When the V_D_ is less than L, LE is indicated and the null hypothesis of panmixia is accepted. When Isoka and Petauke samples were treated as a single population, a *I*_*A*_^*S*^ value of 0.078 (p< 0.001) and a V_D_ value greater than the value of L was obtained, suggesting LD (Table 3). To test the hypothesis of geographical sub-structuring, the *I*_*A*_^*S*^, V_D,_ and L values were calculated separately for samples from each district. The overall *I*_*A*_^*S*^ value for Isoka district was 0.104 (p< 0.001) and the V_D_ value was greater than the L Value, indicating a state of LD. The *I*_*A*_^*S*^ values for Kanyelele and Kalembe populations within Isoka were 0.075 (p< 0.001) and 0.243 (p< 0.001), respectively, and both areas showed a V_D_ value greater than the L value, suggesting LD (Table 3). An overall *I*_*A*_^*S*^ value of 0.027 (p< 0.015) and a V_D_ value greater than the L value indicating LD were obtained for the Petauke population.

### Multiplicity of infection

The multiplicity of infection for Isoka district was 2.16 while that of Petauke district was 2.44. Kanyelele and Kalembe areas of Isoka district showed multiplicity of infection of 2.22 and 2.06, respectively (Table 4).

**Table 4:** Multiplicity of infection in Petauke and Isoka districts

	**N**	**Mean**	**SD**	**Minimum**	**Maximum**
Petauke district					
Petauke overall	28	2.44	0.44	1.22	3.77
Isoka district					
Kanyelele	19	2.22	0.26	1.77	3.00
Kalembe	14	2.06	0.15	1.88	2.33
Isoka overall	33	2.16	0.23	1.77	3.00
Petauke and Isoka districts (overall)	61	2.28	0.36	1.22	3.77

SD: Standard deviation.

## Discussion

In order to establish effective control measures as well as to assess the effectiveness of the current control measures, information on the population structure of *T. parva* with regard to its epidemiology is important. In this study, we analyzed samples from Kanyelele and Kalembe areas in Isoka district (NP) and from Saukani area in Petauke district (EP) of Zambia, which are approximately 600 km (straight line distance) apart and are separated by the North Luangwa, South Luangwa and Lukusuzi National Parks (Figure 1). To achieve this, we employed micro-satellite analysis, an effective way of studying population structures of a wide range of species. Micro-satellite analysis enables direct genotyping of parasite isolates directly from host blood samples by using specific primers. Recently, a panel of polymorphic micro- and mini-satellite markers for *T. parva* was identified [32-34]. A panel of 16 of these microsatellite markers was initially chosen and only 9 were used in the final analysis (Table 1). All the markers (locus pairs) on the same chromosome were over 182 kbp apart. It is therefore unlikely that any of the markers could have been physically linked. To perform the population genetic study of *T. parva*, a haploid organism, an MLG was constructed for each sample, by assigning a single predominant allele for each marker at each locus for each sample. This method of selecting the most predominant allele at each locus from mixed infections is currently fairly standard although it has limitations [18,21,25]. There are several potential short comings of selecting predominant alleles from mixed populations to form an MLG: (i) each particular marker does not always amplify the predominant allele from all strains (ii) the same strain is not always amplified by a particular marker in all the samples, (iii) target regions in different strains that have the highest sequence homology with markers are easily amplified whether they are representative of the predominant strain or not and (iv) all markers in the selected panel of markers most likely do not amplify the same strain in different samples. To avoid these short falls in mixed infections would involve cloning out samples or to only chose samples with only a single allele and then proceed with analysis and generation of an MLG. In this study however, we used a quantitative method to generate an MLG and by so doing, we assume that this MLG presented the closest representative of the dominant strains across the whole sample size.

Allele frequencies at each locus from the Isoka and Petauke populations showed a high number of unique alleles in each population (Figure 2). The data obtained using markers MS48 and MS53 indicated the possibility of genetic differences between Isoka and Petauke populations as evidenced by the complete lack of shared alleles at these 2 sites (Figure 2A). The separation of alleles on loci MS48 and MS53 might also be due to artifacts and as such the data on these loci should be treated with caution; however analysis of the overall data with and without loci MS48 and MS53 produced similar results (data not shown). The increased number of unique alleles versus shared alleles observed when Isoka and Petauke populations were treated as a single population (Figure 2B) suggests a state of genetic and geographical sub-structuring. Genetic and geographical sub-structuring was also observed on PCA where samples from Isoka and Petauke districts clustered separately (Figure 3A). A few samples (n=3) from Isoka district occupied the same quadrants as those from Petauke (Figure 3A). This state of genetic and geographical sub-structuring indicated by allele frequencies (Figure 2C) and PCA (Figure 3A) was further confirmed by the significant differentiation (F_ST_ = 0.178) observed between the population from Isoka and Petauke districts. Furthermore, a state of LD with a *I*_*A*_^*S*^ value greater than 0 was also observed when the Isoka and Petauke populations were treated as a single population (Table 3), indicating the absence of random mating between *T. parva* from the 2 populations and consolidating the state of genetic and geographical sub-structuring. The population from Isoka exhibited less diversity than that from Petauke. These observations therefore, suggests that the parasite populations from these 2 areas, while comprising a similar number of genotypes per locus, exist as separate populations because there is little or no movement of animals between these 2 districts, which in turn is caused by their separation by physical geographical barriers i.e. the national parks (Figure 1). These findings are in agreement with a study on a *T. parva* population wherein geographical sub-structuring was observed between the population from 2 areas separated by a distance of 300 km and a lake [18].

The population from Kanyelele occupied all four quadrants while that from Kalembe only occupied three quadrants (Figure 3B). Although the Kanyelele population was more diverse than the Kalembe population in Isoka district, only moderate genetic differentiation (F_ST_ = 0.049) was observed between the 2 populations (Table 3). This was indicative of the existence of similar genotypes of *T. parva* in both areas. However, the ability of the parasites to randomly mate was restricted as shown from the state of LD, both at the district level and within each sub-population (Table 3). In the Petauke population, significant genetic differentiation (F_ST_ = 0.154) and a state of LD was also observed. This state of LD in Isoka (overall and in sub-populations) and Petauke might be due to the lack of mixing or movement of animals resulting in a lack of gene flow between the populations that can likely be attributed to the relative isolation of the sample areas from each other and the resulting restricted circulation of genotypes. Despite Kanyelele and Kalembe being far apart, animals from the two areas tend to share grazing grounds during the dry period because of the scarcity of pastures and thus it can be hypothesized that the sharing of grazing grounds allows the introduction of genotypes indigenous to one area into another area. However, due to the availability and close proximity of moderate vegetation for animal grazing during the month of May and the preceding months, it is unlikely that animals from Kanyelele and Kalembe areas would have mixed or shared grazing grounds as they will tend to graze pastures closer to their villages. This lack of mixing of animals would have prevented the introduction of genotypes from one area into another and vice-versa, hence the state of restrictive circulation. The tick vector population is also low at this particular time of the year, thus reducing the challenge levels of infection (i.e. low levels of transmission). It can further be hypothesized that the low challenge levels of infection is also likely to give rise to the presence of fewer genotypes of *T. parva* which may result in the state of LD and non-panmixia observed in the majority of areas/regions. This is in agreement with a report in which *Plasmodium falciparum* populations showed strong LD, extensive population differentiation and low diversity in regions of low transmission [26]. The state of LD in these populations might also be due to the presence of epidemic strains [17]. A large sample size, coupled with tick vector ecology data of a wider geographical area in Petauke and Isoka districts, is required to completely validate the LD states observed in this study.

The majority of samples analyzed in this study comprised mixed infections, with Petauke district showing a higher multiplicity of infection than Isoka district (Table 4). Within Isoka district, Kanyelele area also showed a higher multiplicity of infection than Kalembe area (Table 4). Several reasons may be advanced for this situation including (i) transmission intensity reflected by the tick burden on cattle, (ii) the level of infection of the parasite in the ticks, (iii) cattle host factors such as age and breed, (iv) farming systems and (v) the geography of the respective regions. However, this data was inconclusive and as such the effect of these factors could not be further investigated.

## Conclusion

The results of this study indicate that the *T. parva* population from Isoka district is distinctively different from that of Petauke district, with significant gene diversity in each population. There was no evidence of genetic exchange among the populations from the 2 districts. Additionally, these results show that the populations from Kanyelele and Kalembe areas in Isoka district comprised similar genotypes although panmixia of parasites between the 2 areas could not be demonstrated. The sub-structuring and population structure of the *T. parva* populations observed in each district could be due to the restrictive circulation of parasites as a result of the lack of mixing of animals from different regions and/or the low transmission levels of *T. parva* in the respective areas within each district, which may be due to the reduced population of tick vectors at the time of sampling.

## Competing interests

The authors declare no competing interest.

## Authors’ contributions

WM performed the molecular genetic analyses, data analysis, and statistical analysis and drafted the manuscript. BN was involved in field collection and helped to draft the manuscript. RN purified DNA from the parasite reference stocks and helped in editing the manuscript. MS participated in analyzing data and drafting the manuscript. TK and KI participated in drafting the manuscript. NI helped in collecting the samples and editing the manuscript. CS and HS helped to conceive the study, participate in its design and assisted in obtaining funding. All the authors read and approved the final manuscript.
